# 

**Published:** 1899-11-25

**Authors:** 


					THE HOSPITAL. "The standard of highest, purity,"?Lancet. Novembkr 25th, 1399.
CADBURY'S r?* H "fc* CADBURY'S
COCOA lb COCOA
ABSOLUTELY PURE. ^ NO DRUGS OR ALKALIES USED.
No. 687\Aol. XXYIT. [Sspfpen] Saturday,
Nov. 25tii, 1899.
[Subscription Terms,~j pRICE TWOPENCE.

				

